# Supplementary material for: Copper sulfide nanoparticles as a photothermal switch for TRPV1 signaling to attenuate atherosclerosis
Source: Nat Commun. 2018 Jan 15;9:231. doi: 10.1038/s41467-017-02657-z (PMC5768725; doi:10.1038/s41467-017-02657-z)
Supplement: Supplementary file 1 — Supplementary Information [file 41467_2017_2657_MOESM1_ESM.pdf]

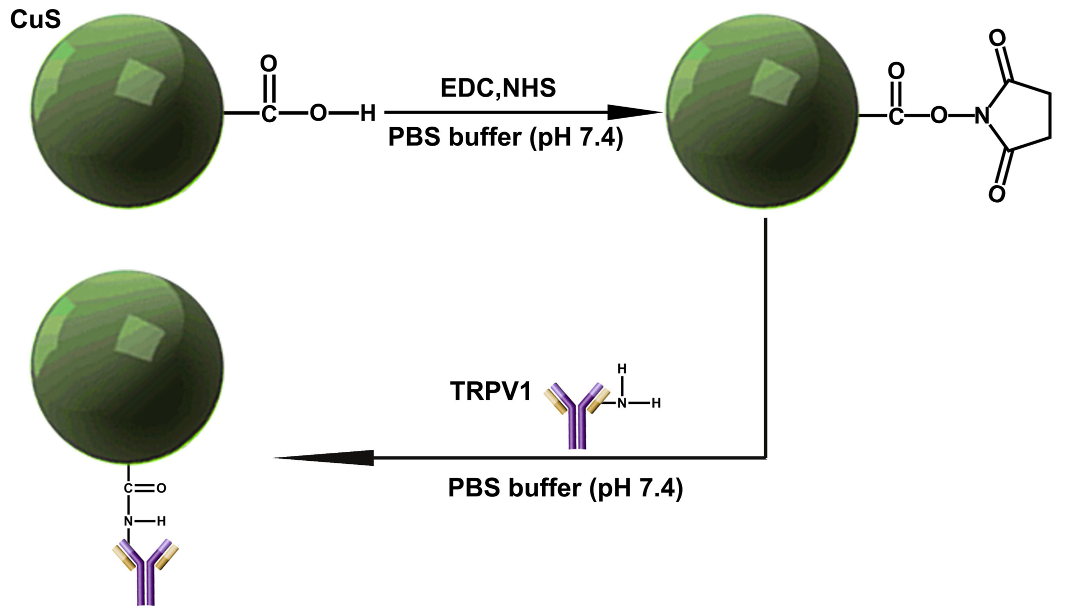


**Supplementary Figure 1.** Schematic of the synthesis of CuS-TRPV1.

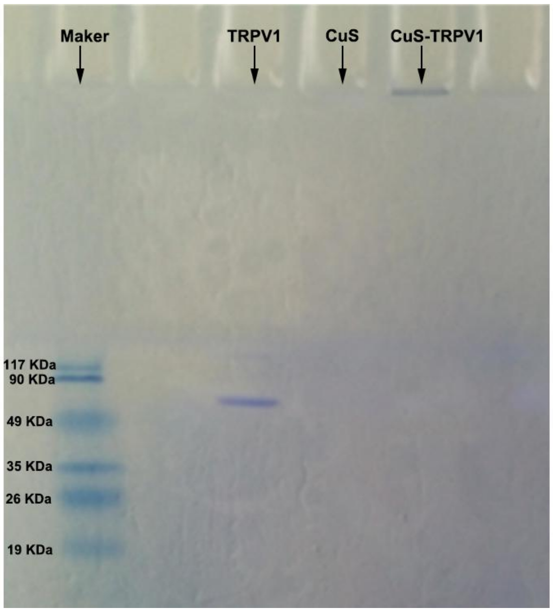


**Supplementary Figure 2.** Gel electrophoresis image of TRPV1, CuS and CuS-TRPV1. TRPV1 (2 μg), CuS (0.4 mg), CuS-TRPV1 (0.4 mg) were incubated with a loading buffer (5×) at 100 °C for 5 min and loaded onto the 12% SDS-PAGE gel. Due to the bulky size of CuS-TRPV1, the band is near the top of the gel.

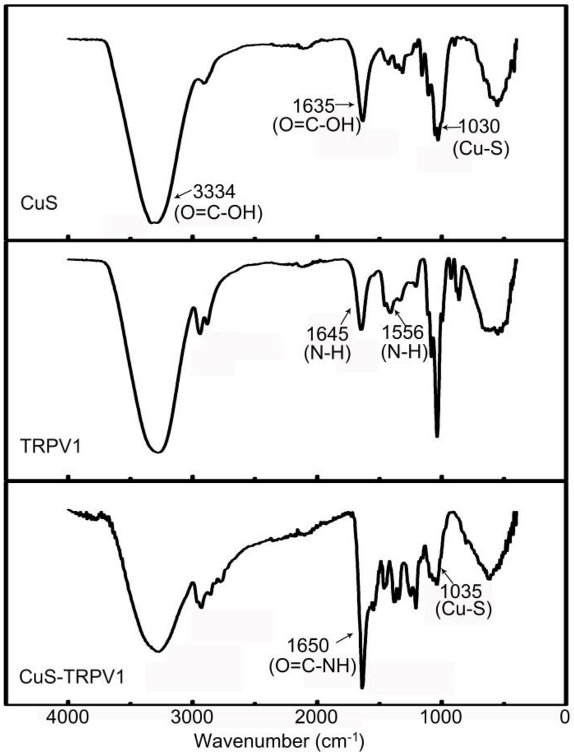


**Supplementary Figure 3.** FT-IR spectra of CuS, TRPV1 antibody and CuS-TRPV1. CuS NPs displayed feature absorption band at 1,030 $cm^{-1}$, which were attributed to Cu-S groups[1]. Peak intensities at 3,334 $cm^{-1}$ and 1,635 $cm^{-1}$ were due to the introduction of COOH groups derived from citrate ligands[2]. TRPV1 displayed strong absorption signals at 1,645 $cm^{-1}$ and 1,556 $cm^{-1}$, which were attributed to the stretching bands of N-H groups. After covalently coupled with COOH groups of CuS NPs, a strong peak at 1,650 $cm^{-1}$ was observed, due to the amide carbonyl stretching[3]. These results suggested that CuS-TRPV1 was successfully synthesized.

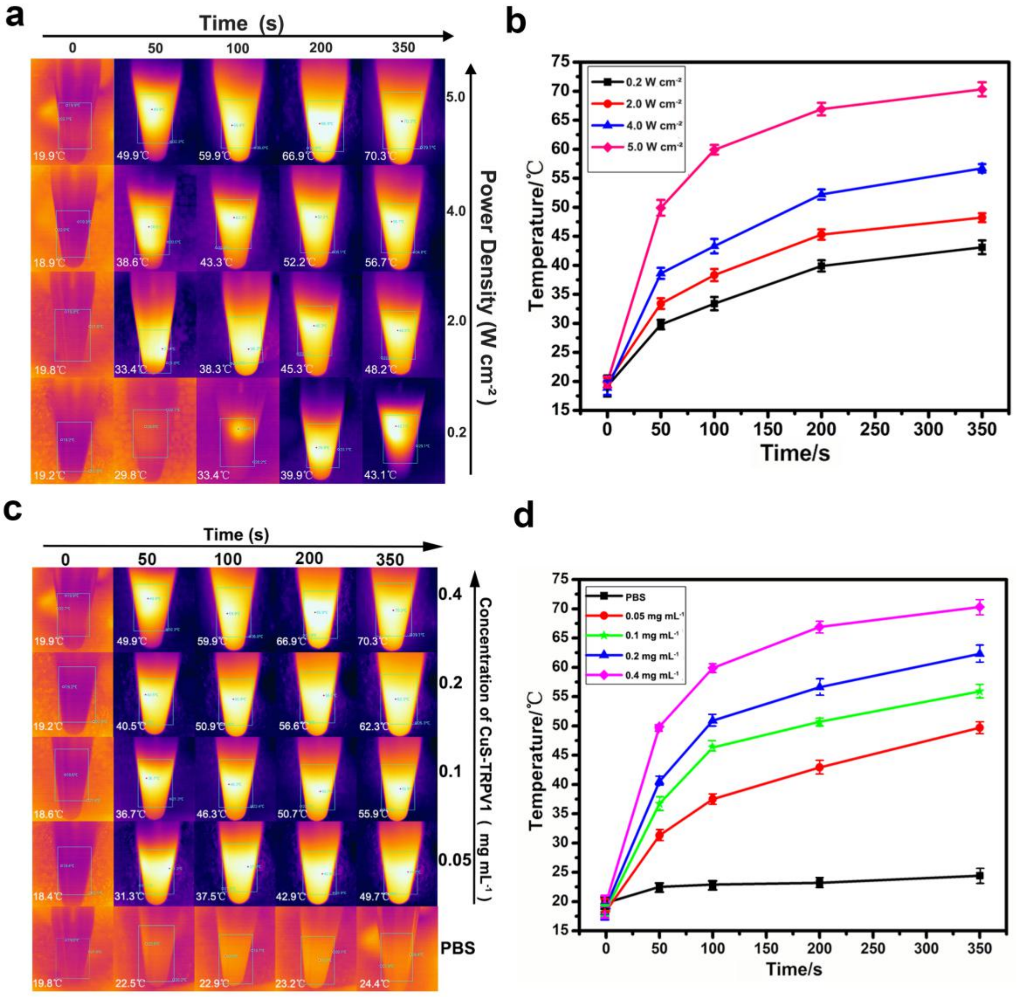


**Supplementary Figure 4.** (a) Real-time thermal images of CuS-TRPV1 at different laser power density. (b) *In vitro* temperature change curves of CuS-TRPV1 at different laser power density. (c) Real-time thermal images of CuS-TRPV1 at different concentrations with laser irradiation for 350 s (5 W $cm^{-2}$). (d) *In vitro* temperature change curves of CuS-TRPV1 at different concentrations with laser irradiation for 350 s (5 W $cm^{-2}$). For all graphs, data are shown as mean ± S.D. of three independent experiments.

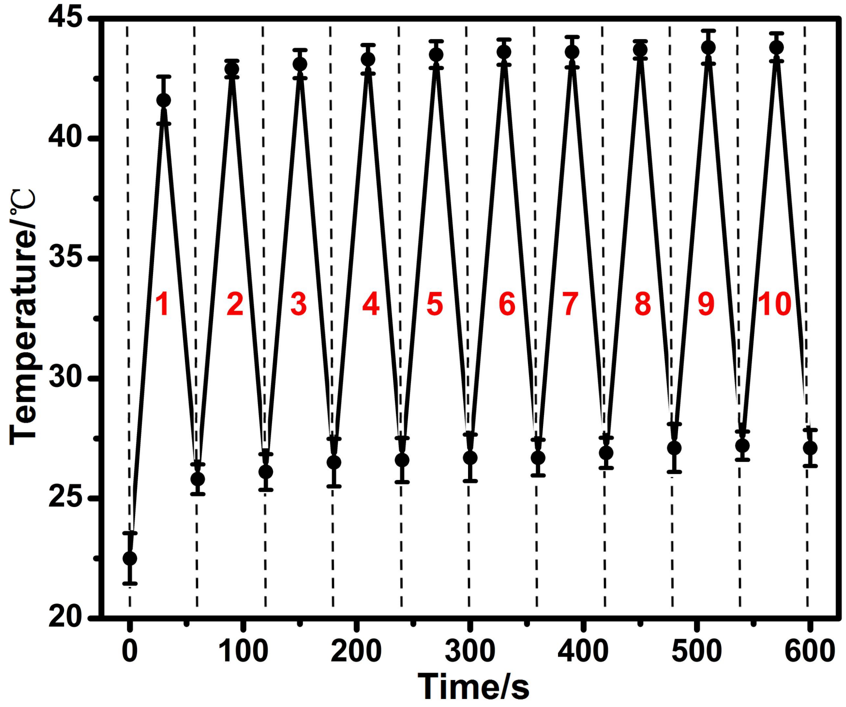


**Supplementary Figure 5**. Transient thermal measurements of CuS-TRPV1 (0.4 mg $mL^{-1}$) under repeated cycles of laser irradiation (980 nm, 5 W $cm^{-2}$). Each cycle consisted of 30 s irradiation followed by a 30 s cooling phase. Data are shown as mean ± S.D. of three independent experiments.

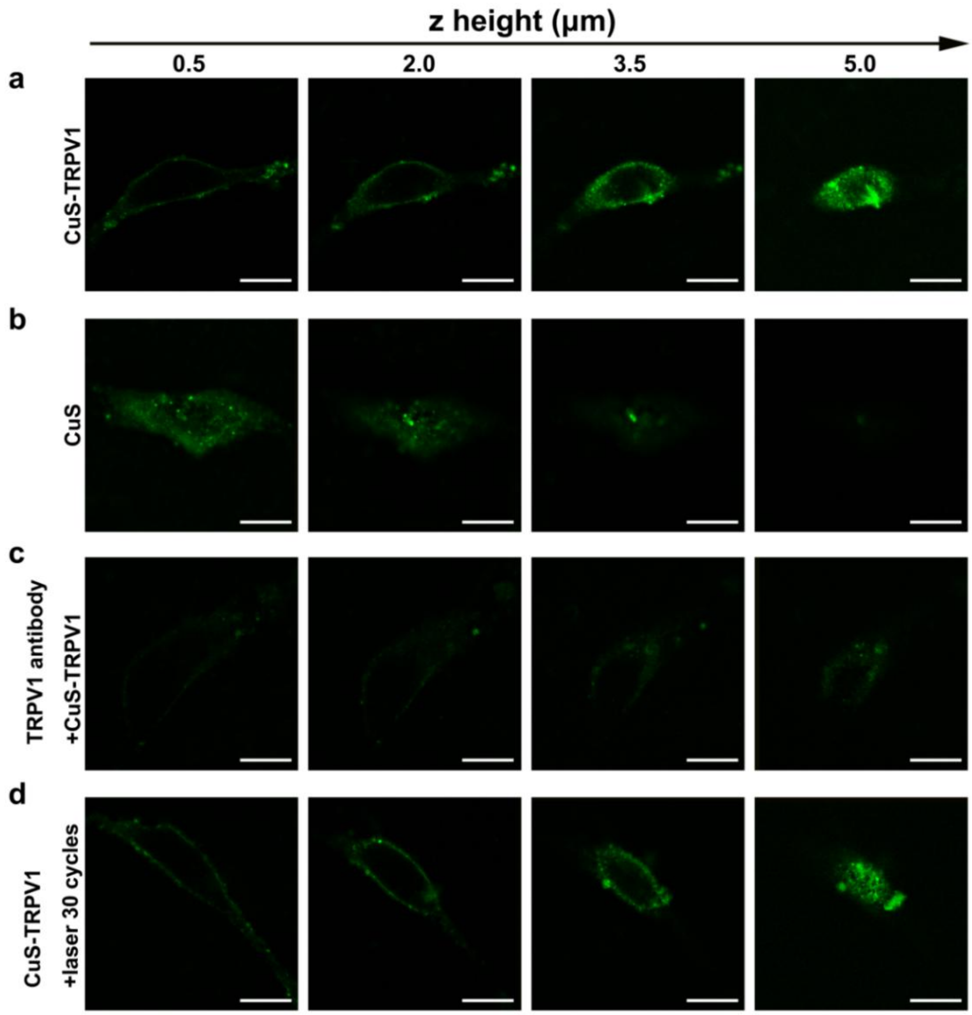


**Supplementary Figure 6.** Representative confocal z-height images of VSMCs (a) incubated with fluorescein-conjugated CuS-TRPV1 (0.4 mg $mL^{-1}$) for 2 h, (b) incubated with fluorescein-conjugated CuS (0.4 mg $mL^{-1}$) for 2 h, (c) pretreated with TRPV1 antibody (0.5 mg $mL^{-1}$) and then incubated with fluorescein-conjugated CuS-TRPV1 (0.4 mg $mL^{-1}$) for 2 h, and (d) incubated with fluorescein-conjugated CuS-TRPV1 (0.4 mg $mL^{-1}$) for 2 h and then irradiated by the 980 nm laser (5 W $cm^{-2}$) for 30 cycles. Images are representative of three independent wells for each group. Scale bar = 5 μm.

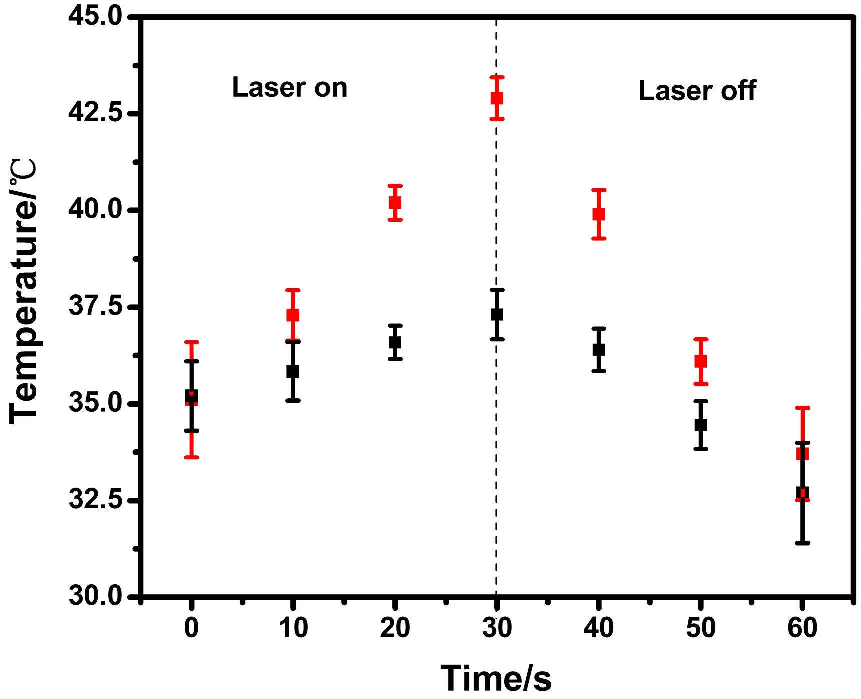


**Supplementary Figure 7.** The local temperature on the VSMCs upon 30 s NIR irradiation followed by a 30 s cooling phase. Black: control group; Red: 0.4 mg $mL^{-1}$ CuS-TRPV1 group. Data are shown as mean ± S.D. of three independent experiments.

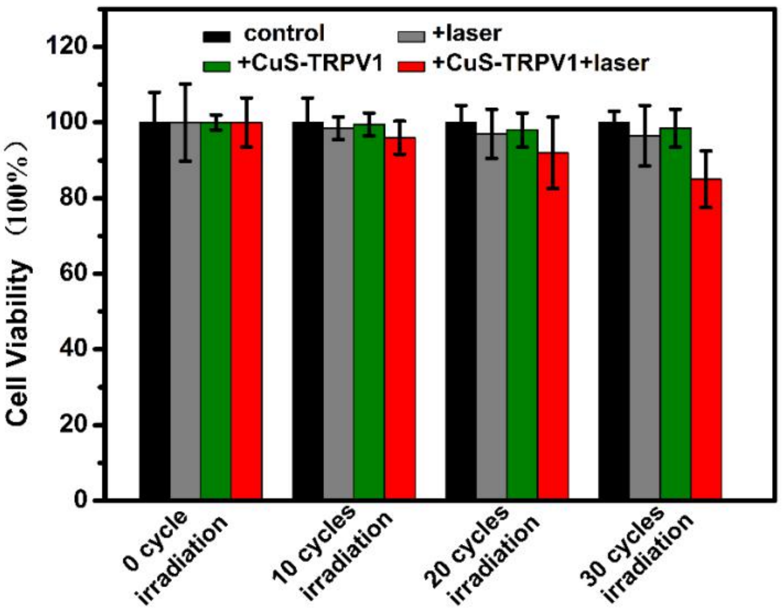


**Supplementary Figure 8.** Cell viability of VSMCs incubated with or without 0.4 mg $mL^{-1}$ CuS-TRPV1 for different laser on/off cycles. Data are shown as mean ± S.D. of three independent experiments, and analyzed by Student's *t*-test. No statistical significance was detected.

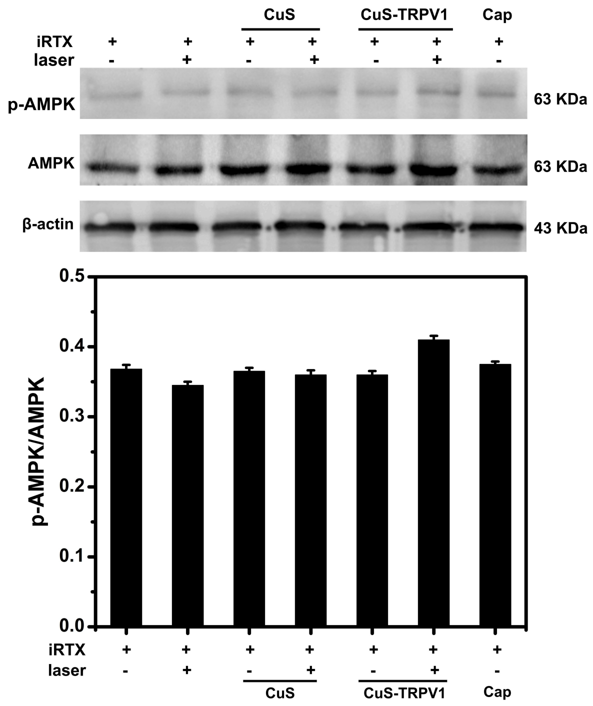


**Supplementary Figure 9.** Western blot analysis of the involvement of TRPV1 in AMPK phosphorylation. VSMCs were incubated with CuS-TRPV1 (0.4 mg $mL^{-1}$) combined with iRTX (1 μM) and irradiated by the 980 nm laser (5 W $cm^{-2}$) for 10 cycles. Capsaicin (Cap, 1 μM) was used as a positive control and CuS (0.4 mg $mL^{-1}$) was used as a negative control. The AMPK phosphorylation was normalized to total AMPK, with β-actin serving as a loading control. Data are shown as mean ± S.D. of three independent experiments, and analyzed by Student's *t*-test. No statistical significance was detected.

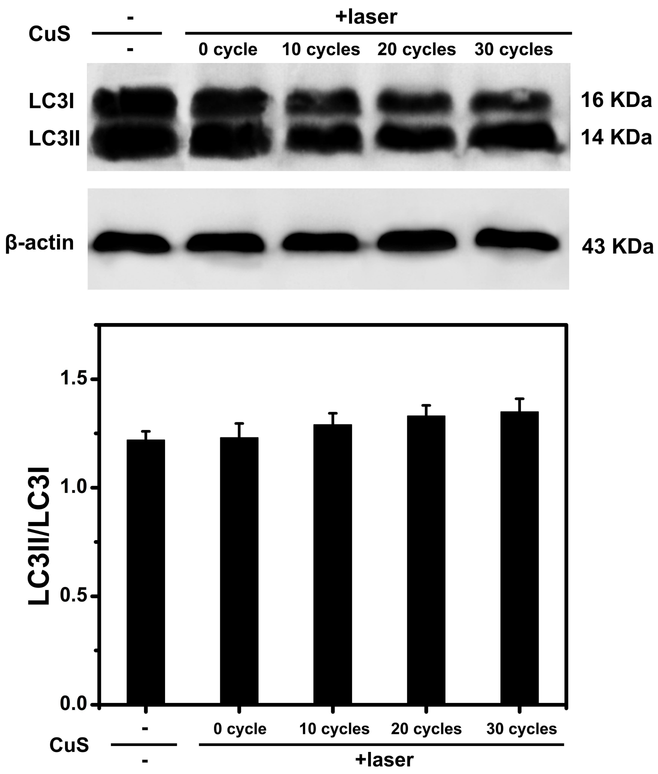


**Supplementary Figure 10.** Western blot analysis of LC3II/LC3I ratio at the indicated irradiation cycle. 80 μg $ml^{-1}$ oxLDL-pretreated VSMCs were incubated with CuS (0.4 mg $mL^{-1}$) and irradiated by the 980 nm laser (5 W $cm^{-2}$) for different cycles (0, 10, 20 or 30 cycles). β-actin was used as a loading control. Data are shown as mean ± S.D. of three independent experiments, and analyzed by Student's *t*-test. No statistical significance was detected.

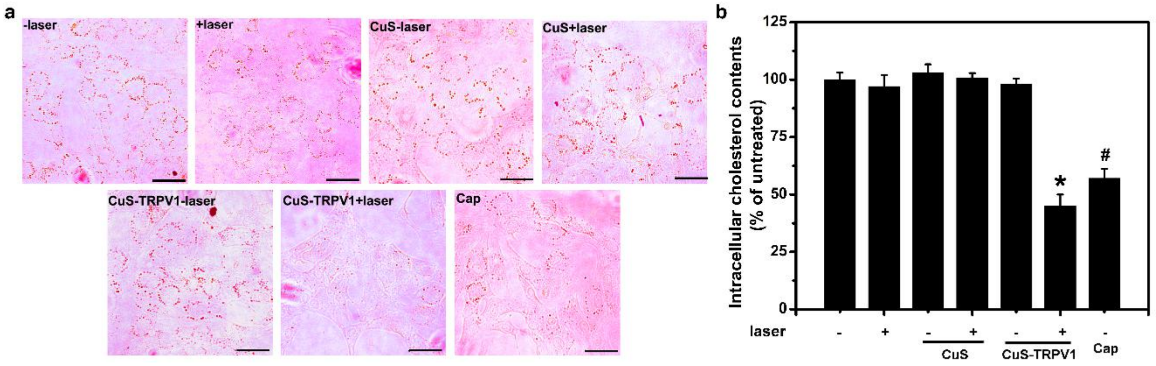


**Supplementary Figure 11.** Photothermal activation of TRPV1 by CuS-TRPV1 reduced lipids in VSMCs. 80 μg $ml^{-1}$ oxLDL-pretreated VSMCs were incubated with CuS-TRPV1 (0.4 mg $mL^{-1}$) and irradiated by the 980 nm laser (5 W $cm^{-2}$) for 30 cycles. Capsaicin (Cap, 1 μM) was used as a positive control and CuS (0.4 mg $mL^{-1}$) was used as a negative control. (a) Representative pictures of Oil red O staining of intracellular lipid droplets. Images are representative of three independent wells for each group. Scale bar = 50 μm. (b) Quantitative analysis of total cholesterol levels. Data are shown as mean ± S.D. of three independent experiments and normalized to untreated cells. $^{*}P < 0.05$ for CuS-TRPV1 vs. untreated, $^{\#}P < 0.05$ for Cap vs. untreated.

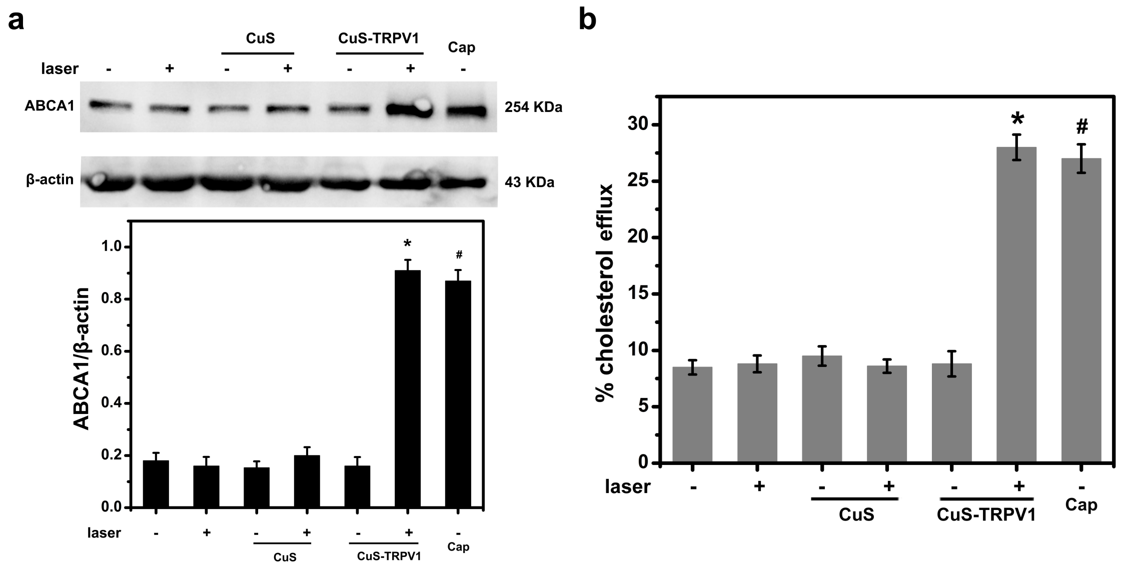


**Supplementary Figure 12**. Photothermal activation of TRPV1 by CuS-TRPV1 upregulated ABCA1 expression and increased cholesterol efflux in VSMCs. OxLDL (80 μg $mL^{-1}$) or fluorescent-labeled cholesterol (5 μM)-pretreated VSMCs were incubated with CuS-TRPV1 (0.4 mg $mL^{-1}$) and irradiated by the 980 nm laser (5 W $cm^{-2}$) for 30 cycles. Capsaicin (Cap, 1 μM) was used as a positive control and CuS (0.4 mg $mL^{-1}$) was used as a negative control. (a) Western blot analysis of ABCA1 expression after indicated treatments. β-actin was used as a loading control. (b) Quantitative analysis of cholesterol efflux after indicated treatments. For all graphs, data are shown as mean ± S.D. of three independent experiments, and analyzed by Student's *t*-test. $^{*}P < 0.05$ for CuS-TRPV1 + laser vs. untreated group, $^{\#}P < 0.05$ for Cap vs. untreated group.

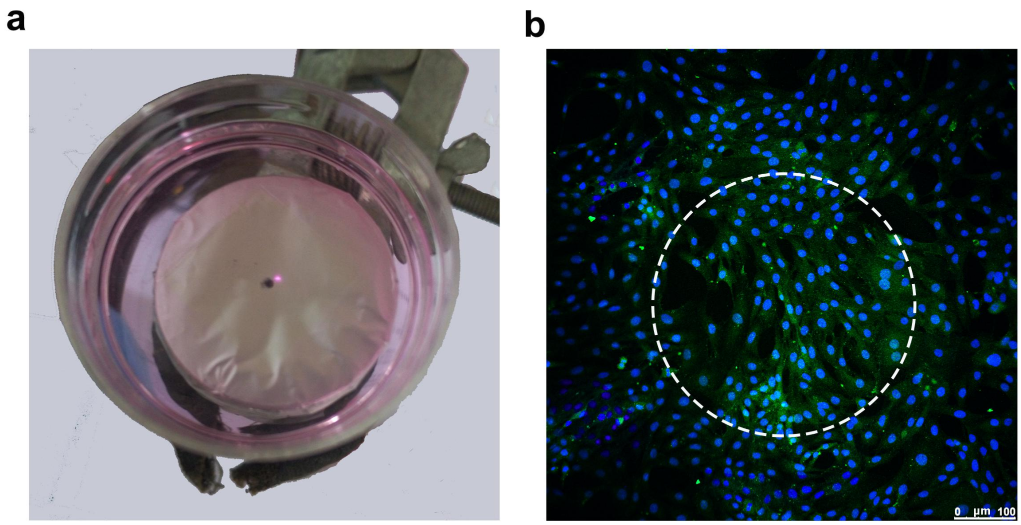


**Supplementary Figure 13.** Spatial control of TRPV1 signaling. CuS-TRPV1 (0.4 mg $mL^{-1}$) are applied to a cell culture slide containing VSMCs. (a) NIR laser focused on the cell culture slide (spot size 300 μm in diameter). (b) Representative fluorescence micrographs of the LC3B (green) signal merged with DAPI-stained nuclei (blue). The dashed line shows the location of laser spot. Images are representative of three independent wells. Sale bar = 100 μm.

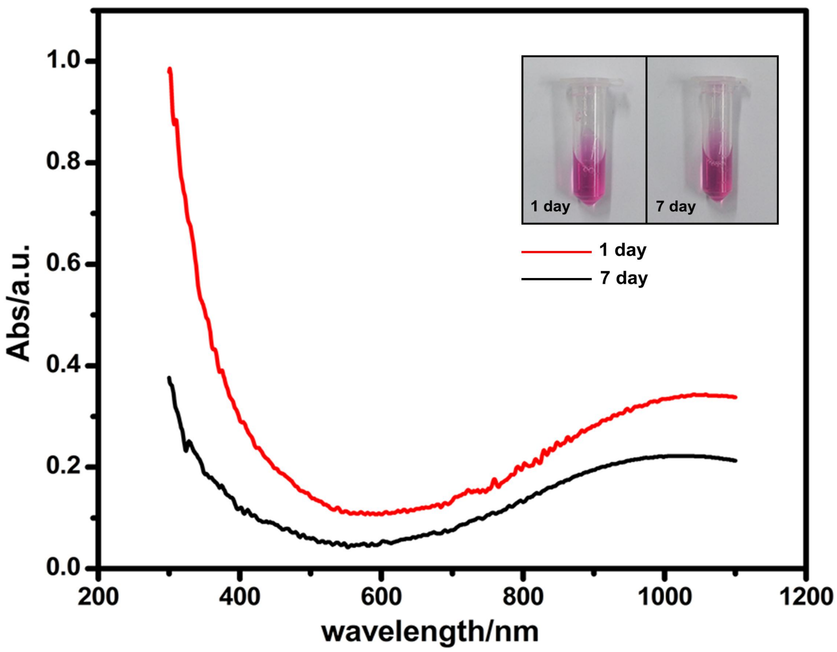


**Supplementary Figure 14.** NIR absorption and stability of CuS-TRPV1 in serum containing medium. No significant decrease and precipitation was observed 7 days after incubation at 37 °C. n = 3 independent experiments.

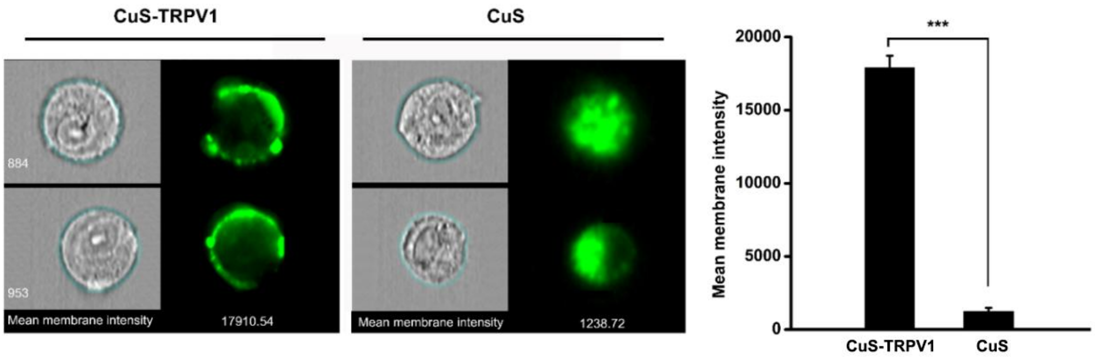


**Supplementary Figure 15.** Localization of CuS-TRPV1 and CuS in VSMCs after 24 h incubation. Cells were incubated with fluorescein-conjugated CuS-TRPV1 or CuS (0.4 mg $mL^{-1}$) for 24 h and visualized by IFC. Much less membrane intensity was observed in VSMCs incubated with CuS (blue area: 1,238.72) than that with CuS-TRPV1 (blue area: 17,910.54). CuS-TRPV1 content decreased over time (63% that of 2 h incubation) as the cellular internalization. Data are shown as mean ± S.D. of three independent experiments, and analyzed by Student's *t*-test. $^{***}P < 0.001$ for CuS-TRPV1 vs. CuS.

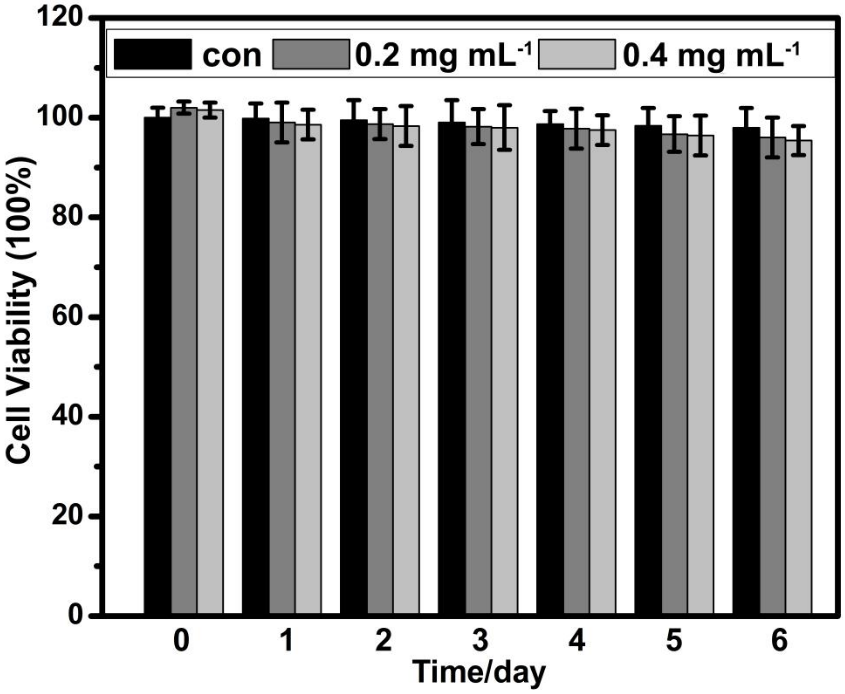


**Supplementary Figure 16.** Long-term cytotoxicity of CuS-TRPV1 on VSMCs. The cells were incubated with different amounts of CuS-TRPV1 (0.2 mg $mL^{-1}$ and 0.4 mg $mL^{-1}$) for 0-6 days, respectively. Cell viability was measured by the MTT assay. Data are shown as mean ± S.D. of three independent experiments, and analyzed by Student's *t*-test. No statistical significance was detected.

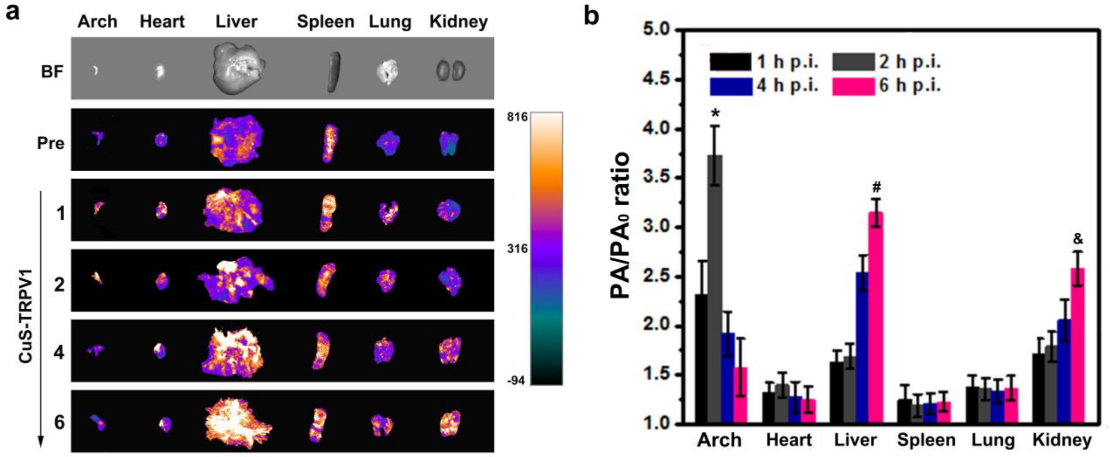


**Supplementary Figure 17.** Biodistribution analysis of CuS-TRPV1 by PA imaging. (a) *Ex vivo* PA images of aortic arch and major organs excised at 1, 2, 4 and 6 h post-injection. (b) Quantification of PA signals in aortic arch and each organ. Compared with PA signals of pre-injection, the increased PA signals at indicated time points were calculated. Data are shown as mean ± S.D. (n = 3), and analyzed by Student's *t*-test. $^{*}P < 0.05$ vs. pre in aortic arch, $^{\#}P < 0.05$ vs. pre in liver, $^{\&}P < 0.05$ vs. pre in kidney.

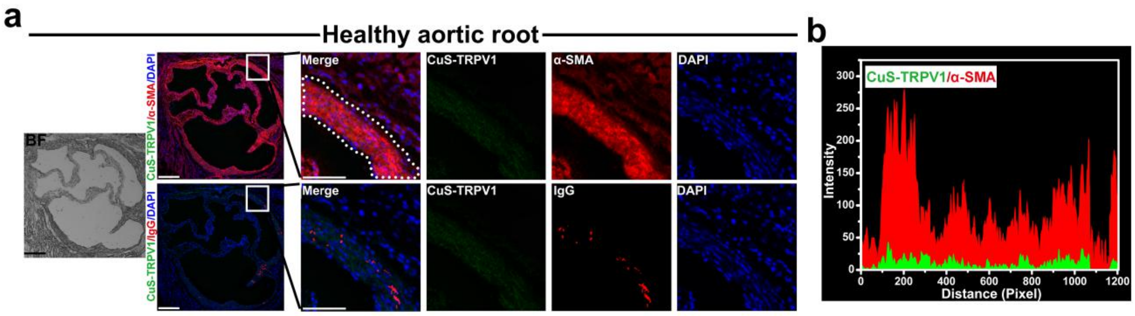


**Supplementary Figure 18.** (a) Representative immunofluorescent micrographs of aortic root sections showing CuS-TRPV1 accumulation in healthy aortas at 2 h post-injection. From right, blue (DAPI), red (VSMC maker α-SMA or background control IgG), green (fluorescein-conjugated CuS-TRPV1) and merge. The white square gave a view of the magnification of a region of atherosclerotic lesion as indicated on the right (n = 3 mice per group, Scale bar = 75 μm). (b) Fluorescence intensity profile of the region enclosed by the white dotted line.

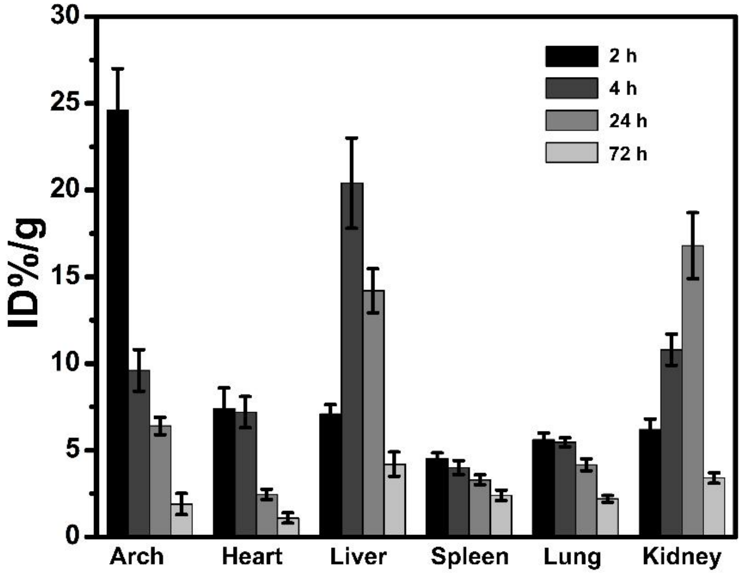


**Supplementary Figure 19.** ICP-AES of Cu content in mice at 2 h, 4 h, 24 h and 72 h after intravenous injection with CuS-TRPV1 (0.4 mg $mL^{-1}$). Data are shown as mean ± S.D. (n = 3)

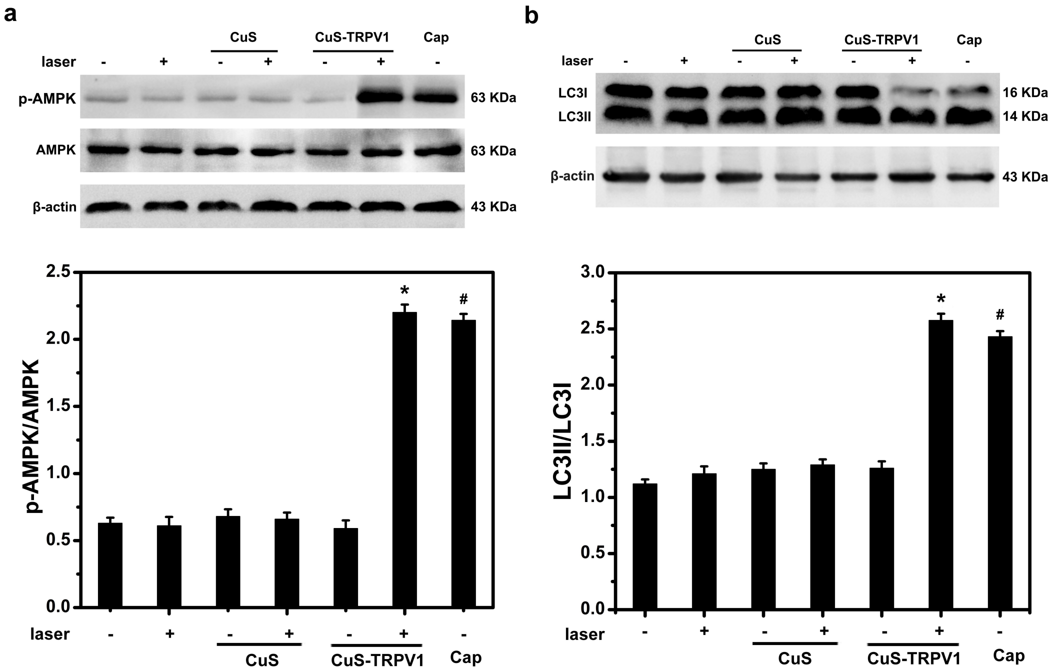


**Supplementary Figure 20.** Western blot analysis of p-AMPK/AMPK and LC3II/ LC3I in the aortic arch lesions from high-fat diet-fed ApoE$^{-/-}$ mice received as indicated treatment. Data are shown as mean ± S.D. (n = 6), and analyzed by Student's *t*-test. $^{*}P <$ 0.05 for CuS-TRPV1 vs. untreated group, $^{\#}P < 0.05$ for Cap vs. untreated group.

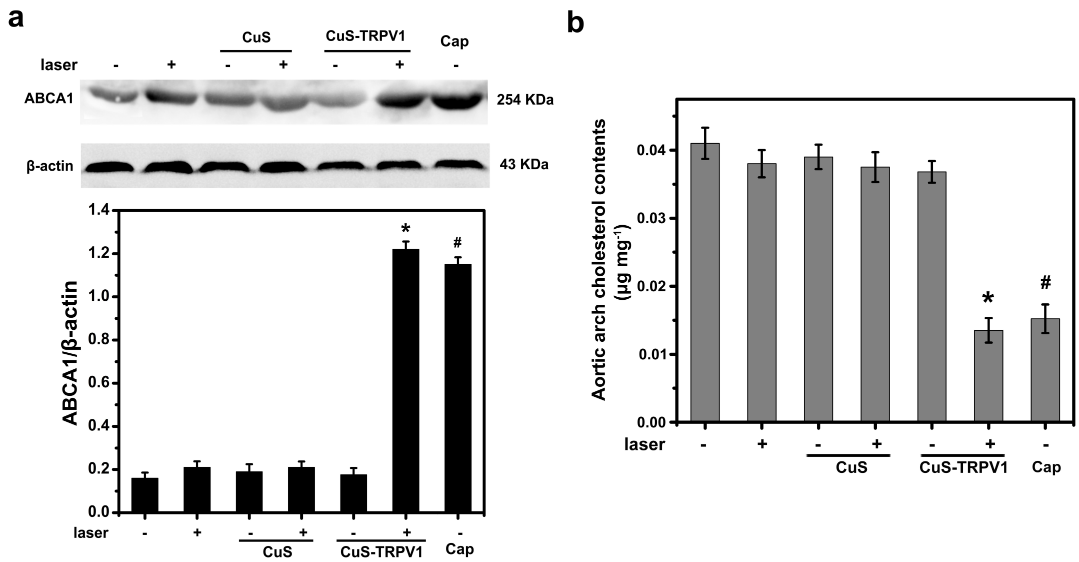


**Supplementary Figure 21.** (a) Western blot analysis of ABCA1 expression and (b) Quantitative analysis of total cholesterol levels in the aortic arch lesions from high-fat diet-fed ApoE$^{-/-}$ mice received as indicated treatment. Data are shown as mean ± S.D. (n = 6), and analyzed by Student's *t*-test. $^{*}P < 0.05$ for CuS-TRPV1 vs. untreated group, $^{\#}P < 0.05$ for Cap vs. untreated group.

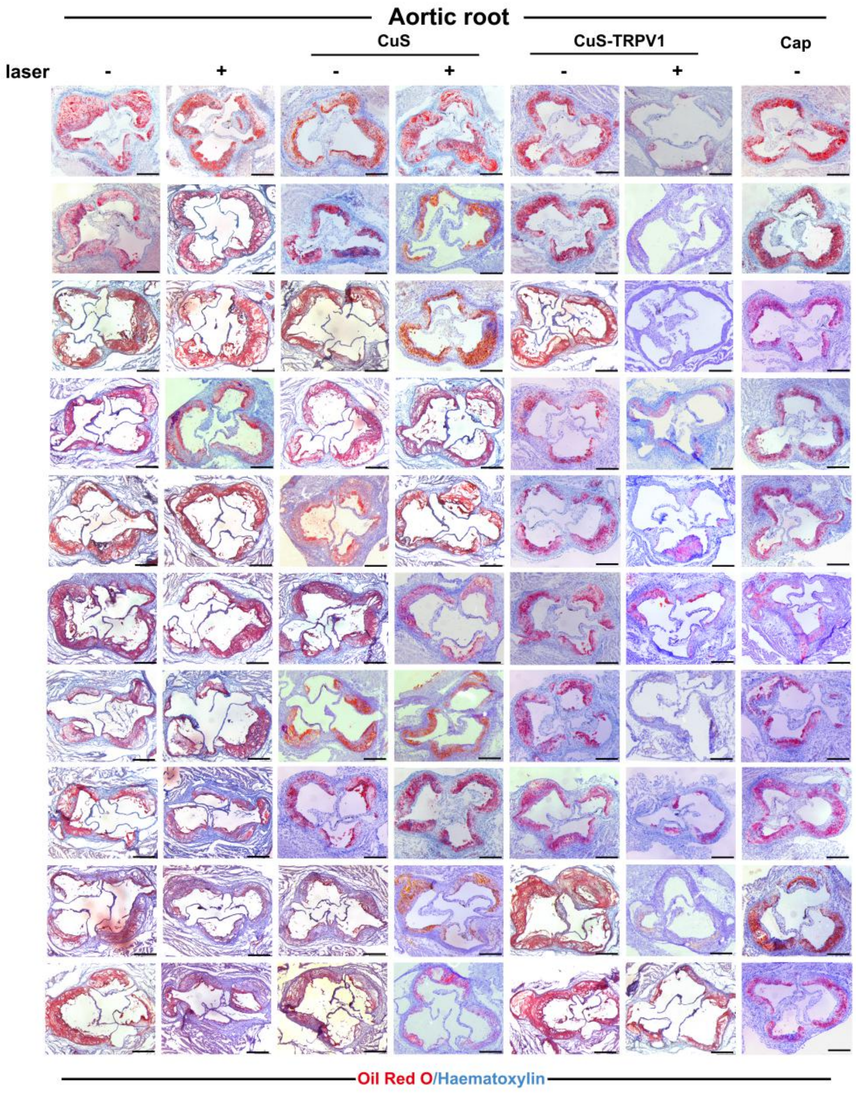


**Supplementary Figure 22.** Oil Red O-stained aortic root sections from treatment (laser; CuS; CuS+laser; CuS-TRPV1; CuS-TRPV1 + laser; Capsaicin) and control (PBS only) groups of mice (n = 10) after 12 weeks. Haematoxylin was used as a counterstain. Scale bar = 250 μm.

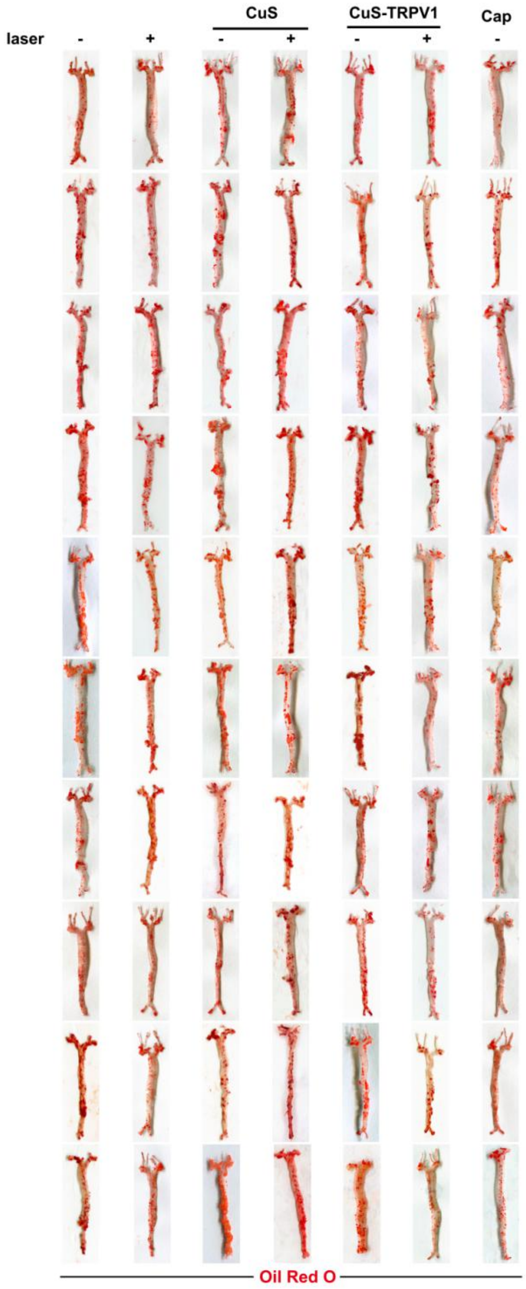


**Supplementary Figure 23.** Oil Red O-stained *en face* aortic preparations from treatment (laser; CuS; CuS + laser; CuS-TRPV1; CuS-TRPV1 + laser; Capsaicin) and control (PBS only) groups of mice (n = 10) after 12 weeks.

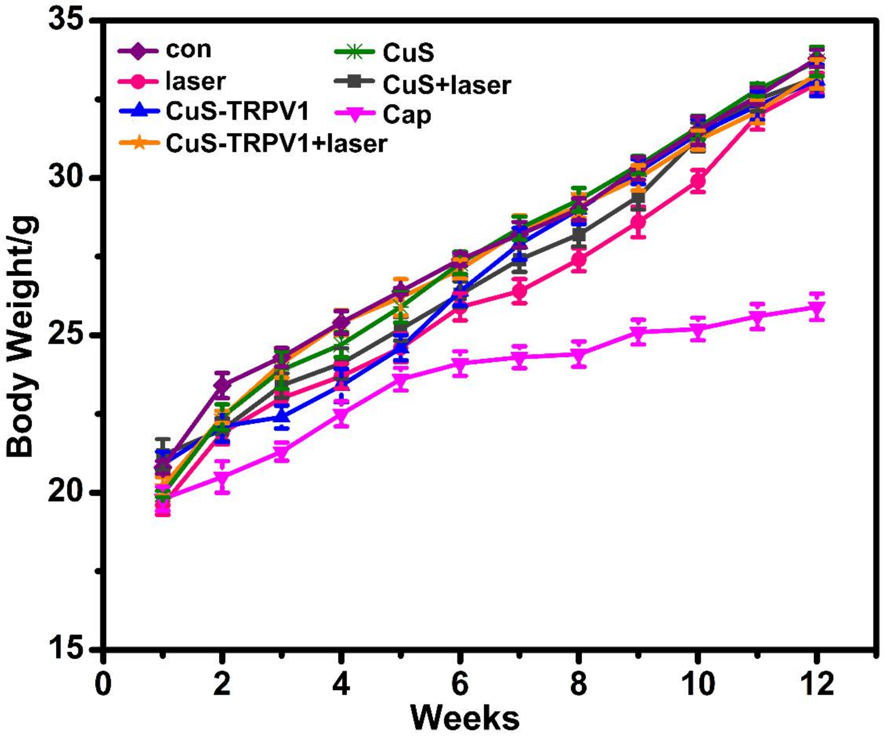


**Supplementary Figure 24.** 12-week growth chart of mice from treatment (laser; CuS; CuS + laser; CuS-TRPV1; CuS-TRPV1 + laser; Capsaicin) and control (PBS only) groups (n = 10).

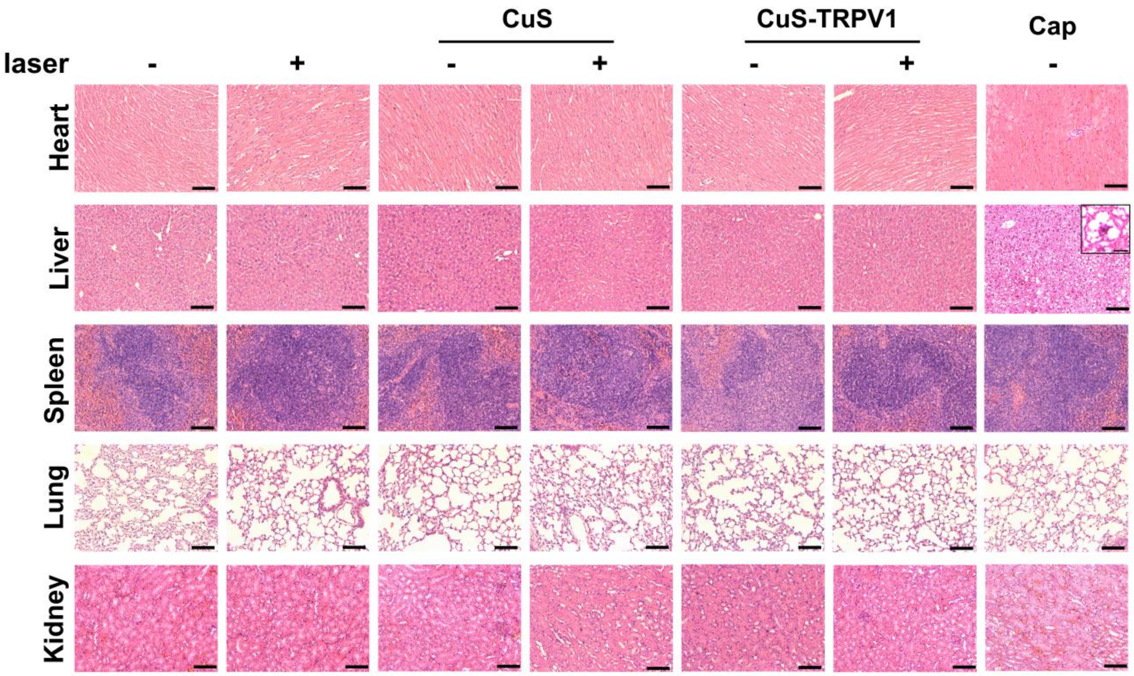


**Supplementary Figure 25.** Representative histology (H&E) images of major organs collected from treatment (laser; CuS; CuS + laser; CuS-TRPV1; CuS-TRPV1 + laser; Capsaicin) and control (PBS only) groups of mice after 12 weeks (n = 10, Scale bar = 100 μm). Inset: magnification of hepatocellular steatosis and shrinkage (Scale bar = 10 μm).

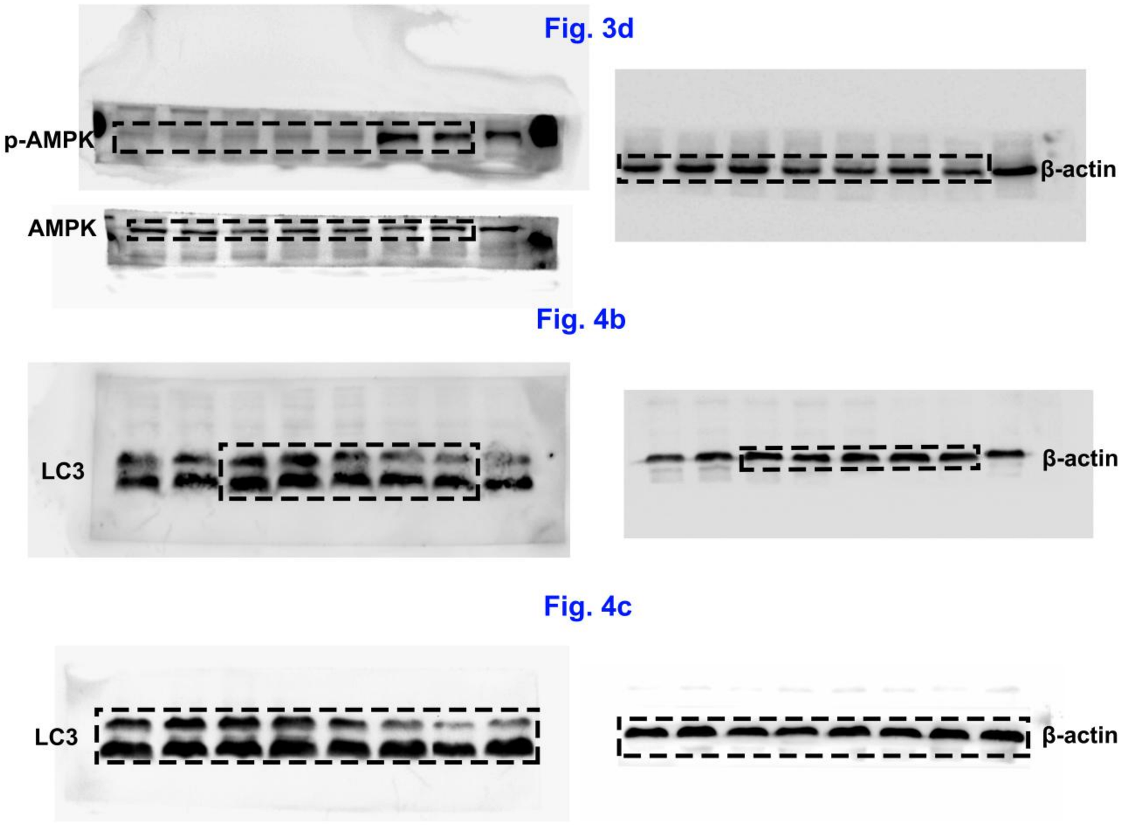


**Supplementary Figure 26.** Uncropped western blot scans used in the main figures. Blots correspond to those shown in Figure 3d, Figure 4b and 4c within the main manuscript.

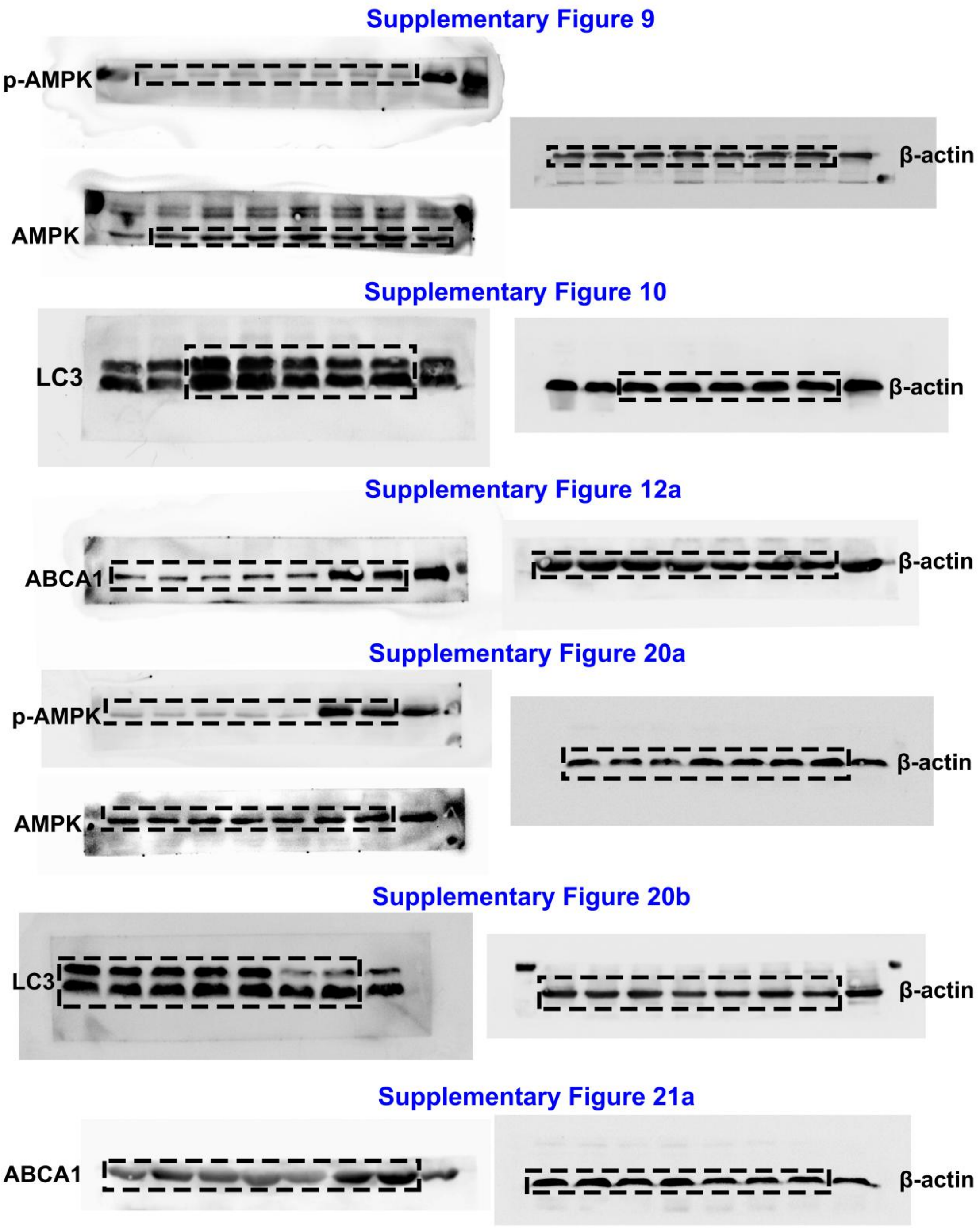


**Supplementary Figure 27.** Uncropped western blot scans used in the supplementary figures. Blots correspond to those shown in Supplementary Figure 9, Supplementary Figure 10, Supplementary Figure 12a, Supplementary Figure 20a and b, Supplementary Figure 21a.

**Supplementary References**


1. Theerthagiri, J. *et al*. Synthesis and characterization of a CuS-$WO_3$ composite photocatalyst for enhanced visible light photocatalytic activity. *RSC Adv.* **5**, 52718-52725 (2015).
2. Ray, C. *et al*. Evolution of tubular copper sulfide nanostructures from copper(I)-metal organic precursor: A superior platform for the removal of Hg (II) and Pb (II) ions. *RSC Adv.* **5**, 12446-12453 (2015).
3. Yu, S. *et al*. Facile room-temperature synthesis of carboxylated graphene oxide-copper sulfide nanocomposite with high photodegradation and disinfection activities under solar light irradiation. *Sci Rep.* **5**, 16369 (2015).
